# Value of a preoperative prognostic nutritional index for the prognostic evaluation of gastric neuroendocrine carcinoma patients

**DOI:** 10.3389/fnut.2023.1043550

**Published:** 2023-07-24

**Authors:** Jiangpeng Wei, Ju Lu, Hanxiang Jia, Xisheng Yang, Xin Guo, Jinqiang Liu, Xiaohua Li

**Affiliations:** Department of Gastrointestinal Surgery, Xijing Hospital, Air Force Military Medical University, Xi'an, Shaanxi, China

**Keywords:** Onodera’s prognostic nutrition index, radical gastrectomy, gastric neuroendocrine cancer, adverse events, overall survival

## Abstract

**Objective:**

To study the value of Onodera’s prognostic nutrition index (PNI) in patients with gastric neuroendocrine cancer (G-NEC).

**Methods:**

The clinical data on 148 cases of G-NEC presented between March 2010 and April 2022 were retrospectively analyzed. The relationship between the clinical characteristics of the patients and PNI was analyzed. Optimal PNI cutoff values for G-NEC prognosis prediction were calculated using the X-tile software. The survival curves were created using the Kaplan–Meier method. A Cox proportional hazards model was also established to identify independent prognostic factors that impact the prognosis of patients with G-NEC.

**Results:**

The median overall survival (OS) rate was 30 months (range 6–127 months), and the OS rates at 1, 3 and 5 years were 89.2, 71.6 and 68.2%, respectively. The mean PNI of the 148 patients before the operation was 49.5 ± 8.0. The mean PNI of patients with anemia (*p* < 0.001) and abnormal carcinoembryonic antigen (*p* = 0.039) was significantly lower than that of patients without such comorbidities. The mean PNI of patients with Stage III tumors (*p* < 0.001) and postoperative complications was significantly lower (*p* = 0.005). PNI optimal cutoff values were 50 (*p* < 0.001). Based on the cut-off value of the PNI, these patients were divided into a PNI-high group (PNI ≥ 50.0, *n* = 77) and a PNI-low group (PNI < 50.0, *n* = 71). The PNI-high group had a significantly better 5-years OS rate compared with the PNI-low group (76.6% vs. 59.2%, *χ*^2^ = 14.7, *p* < 0. 001). Multivariate analysis demonstrated that PNI and pathological stage were independent prognostic factors for patients with G-NEC. In the subgroup analysis, OS rates were significantly lower in the PNI-low group than in the PNI-high group among patients with stage I and stage III of the disease.

**Conclusion:**

The PNI is a simple and useful marker for predicting long-term outcomes in G-NEC patients regardless of tumor stage. Based on our results, we suggest that PNI should be included in routine assessments of patients with G-NEC.

## 1. Introduction

Despite progress in early detection, surgical techniques, and adjuvant treatment of gastric neuroendocrine carcinoma (G-NEC), this disease is still a health problem worldwide (1). Surgery is the main method of treatment (2). Even after R0 resection has been achieved, certain G-NEC patients still experience postoperative recurrence. Currently, the prognosis of patients with G-NEC is usually determined based on the pTNM stage, but there are still certain defects (such as whether the operation and pathological examination were standardized) in using this method. Therefore, indicators with a higher accuracy rate are needed to determine the prognosis. G-NEC has complex clinical manifestations and its differentiation is closely associated with the endocrine system and metabolism (3). Therefore, these patients require comprehensive multidisciplinary management, with nutritional evaluation of importance for the evaluation and management of these patients (4).

Nutrition and immune status have also been reported to affect the long-term prognosis of patients with malignant tumors (5, 6). Since serum albumin expression is associated with nutritional status and lymphocyte count is associated with immune status, Onodera’s prognostic nutrition index (PNI) can be used to evaluate the nutritional and immune status of patients. Since the 2010s, Onodera’s PNI has been widely used as a predictor of survival in patients with various malignant tumors, including gastrointestinal (7–9) and non-gastrointestinal cancers (10, 11). However, to our knowledge, no study has been conducted to explore the clinical significance and prognostic value of PNI in G-NEC. Therefore, we retrospectively studied the relationship between PNI and clinicopathological factors, as well as the predictive value of PNI for overall survival (OS) in patients with G-NEC.

## 2. Patients and methods

### 2.1. Patients

**Figure 1:**
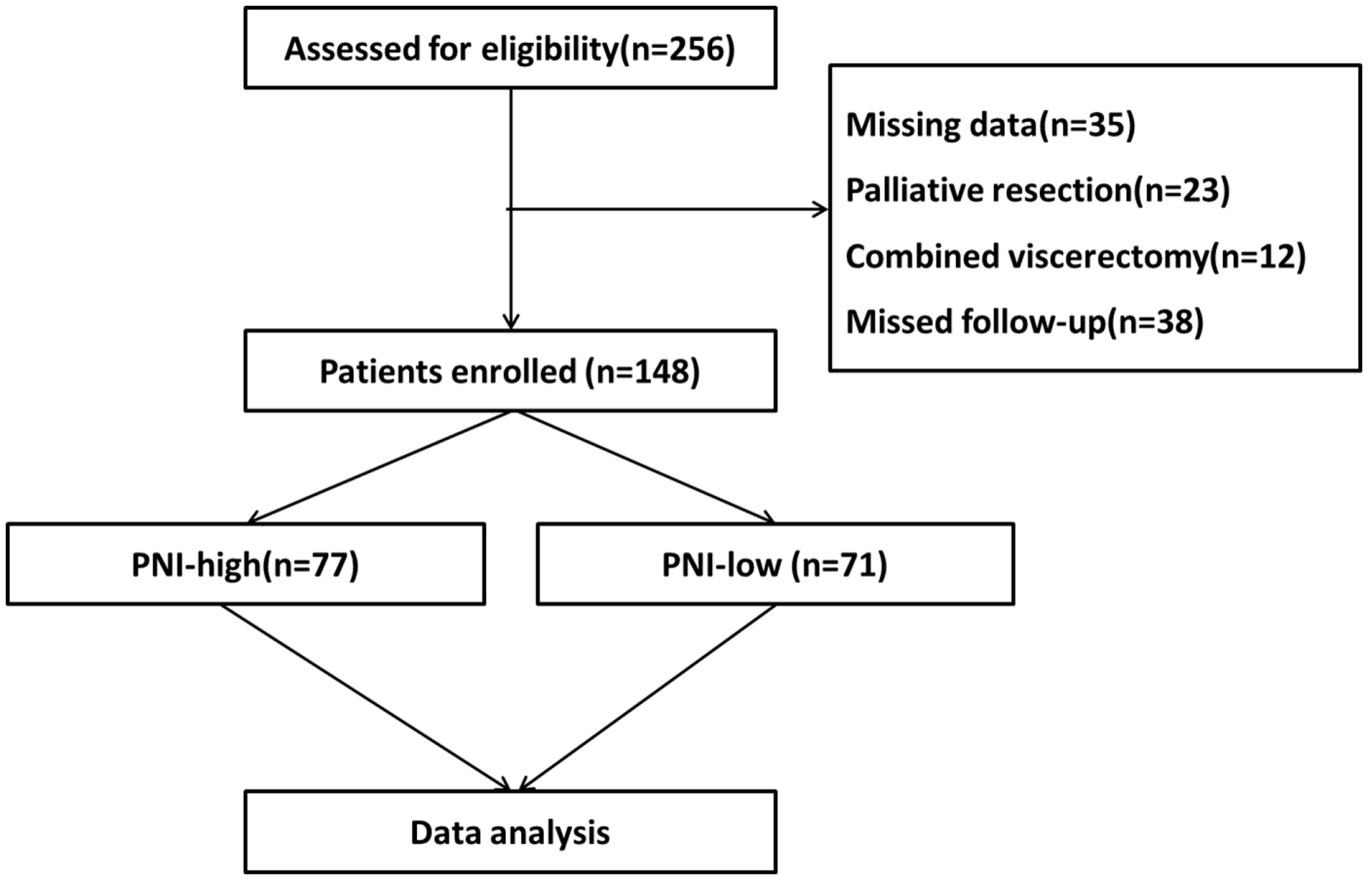
The flowchart of patient selection.

We retrospectively collected clinical data on 148 patients who had undergone radical resection for G-NEC at the Department of Gastrointestinal Surgery of the First Affiliated Hospital of Air Force Military Medical University from March 2010 to April 2022. Inclusion criteria: a pathological diagnosis of G-NEC after surgery; complete clinical and follow-up data; complete radical operation. Exclusion criteria: previous or preoperative use of chemotherapy drugs; incomplete clinical data; poor compliance and treatment not completed as instructed by the doctor (Figure 1). This study was approved by the hospital’s ethics committee and written informed consent was obtained from all patients enrolled for the use of their data.

### 2.2. Data collection

The results of the preoperative blood test, which included the level of serum albumin and total lymphocyte count in peripheral blood, were obtained by reviewing the electronic medical record system of our hospital. The PNI was calculated based on serum albumin (g/L) +5 lymphocyte count (10^9^/L). The basic characteristics of the patients, including age, body mass index (BMI), tumor location, method of operation, tumor depth, lymph node metastasis, postoperative adjuvant therapy, and tumor node metastasis classification (TNM) were also recorded. The incidence of postoperative complications also was evaluated in the present study. The severity of complications was defined according to the Clavien-Dindo classification. The stage of TNM was defined according to the American Joint Committee on Cancer 8th edition. The relationship in PNI, clinicopathological characteristics, postoperative complications, and prognosis was analyzed.

### 2.3. Follow-Up

After discharge, regular telephone interviews or outpatient follow-up visits began 1 month after the operation. In addition to physical examination, follow-up also includes gastroscopy, liver, and lung imaging examination, and serum alpha-fetoprotein (AFP).

Carbohydrate antigen 199 (CA199), carbohydrate antigen 125 (CA125), and carcinoembryonic antigen (CEA) monitoring to determine whether there is distant metastasis and local recurrence. Follow-up was conducted every three months within the first three years and every six months thereafter. Follow-up information on patients was collected from tumor registries and hospital records or was obtained from patients and family members. In our research study, we used OS and disease-free survival (DFS) as the endpoint of the study, as OS is considered the most suitable event for survival analysis. The DFS was defined as the time from the operation to tumor recurrence or death, whichever occurred first. The OS was defined as the time from the operation to death.

### 2.4. Statistical analysis

Count data were summarized using frequencies and percentages and processed using SPSS 22.0 statistical software (Version 22.0, IBM, New York). The appropriate cut-off points of PNI for the prediction of the prognosis of G-NEC were calculated using X-tile software. The PNI and clinicopathological characteristics were analyzed using the Chi-square test or Fisher’s exact test. The Kaplan–Meier method and the log-rank test were performed to compare OS between groups. Significant prognostic risk factors identified through a univariate analysis were further assessed through a multivariate analysis using Cox’s proportional hazards regression model. Hazard ratio (HR) and 95% confidence interval (95% CI) were used as correlation measurements in our research study. A value of p of <0.05 was considered to indicate statistical significance.

## 3. Results

### 3.1. Characteristics of the study population

A total of 148 patients were included in this study. The baseline characteristics of the enrolled patients are shown in Table 1. The mean age of the patients enrolled was 60.0 years and 84.5% of the patients were male. The average PNI of the patients before the operation was 49.5 ± 8.0, while the average PNI of patients ≥65 years old was 46.8 ± 8.0, and the average PNI of patients <65 years old was 50.9 ± 7.6. The difference between the two groups was statistically significant (*p* = 0.003). The mean PNI of patients with Stage III of the disease was significantly lower than that of Stage I and II patients (*p* = 0.046). The mean PNI of patients with anemia (*p* < 0.001) and abnormal CEA (*p* = 0.039) was significantly lower than those without such comorbidities. The mean PNI of patients with postoperative complications was lower than that of patients without postoperative complications (*p* = 0.005). In the subgroup analysis, patients with Clavien I-II complications (*p* = 0.001) and infectious (*p* = 0.001) had a lower mean PNI (Table 1).

**Table 1:** The relationship between the clinicopathological factors and the PNI values are expressed as means and standard deviations.

Clinicopathological		*N* (%)	PNI	Statistical value	*p* value
Sex	Male	125 (84.5)	49.5 ± 8.1	*t* = −0.252	0.801
	Female	23 (15.5)	49.9 ± 7.5		
Age (years)	<65	98 (65.1)	50.9 ± 7.6	*t* = 3.018	0.003
	≥65	50 (34.9)	46.8 ± 8.0		
BMI (Kg/m^2^)	<18	10 (6.8)	47.0 ± 4.3	*t* = −1.036	0.302
	≥18	138 (93.2)	49.7 ± 8.2		
Tumor location	upper	63 (42.6)	50.0 ± 7.7	*χ2* = 0.583	0.559
	lower-middle	56 (37.8)	49.7 ± 7.8		
	Mixed	29 (19.6)	48.1 ± 8.9		
Operation mode	Open	122 (82.4)	49.7 ± 8.1	*t* = 0.386	0.700
	Laparoscopy	26 (17.6)	48.9 ± 7.4		
CEA (ng/ml)	<5	117 (17.6)	50.2 ± 7.9	*t* = 2.087	0.039
	≥5	31 (17.6)	46.9 ± 7.5		
AFP (ng/ml)	<7	130 (87.8)	49.7 ± 8.0	*t* = 0.574	0.567
	≥7	18 (12.2)	48.5 ± 8.0		
CA199 (U/ml)	<18	131 (88.5)	49.8 ± 7.9	*t* = 1.225	0.223
	≥18	17 (11.5)	47.3 ± 8.4		
CA125 (U/ml)	<18	134 (90.5)	49.9 ± 7.7	*t* = 1.944	0.054
	≥18	14 (9.5)	45.6 ± 9.7		
WBC count (*109/L)	Normal	98 (66.2)	50.2 ± 8.0	*t* = 1.463	0.146
	Abnormal	50 (33.8)	48.2 ± 7.8		
Hypohemia	Yes	43 (29.1)	51.1 ± 7.9	*t* = 3.757	<0.001
	No	105 (70.9)	45.9 ± 6.9		
ASA score	I	122 (82.4)	49.9 ± 8.1	*t* = 1.237	0.218
	II-III	26 (17.6)	47.8 ± 7.4		
Tumor depth	T1, T2	33 (22.3)	50.8 ± 6.5	*t* = 1.006	0.316
	T3, T4	115 (77.7)	49.2 ± 8.3		
Lymph node metastasis	Negative	57 (38.5)	49.4 ± 6.7	*t* = −0.223	0.824
	Positive	91 (61.5)	49.7 ± 8.7		
Pathological stage	Stage I, II	105 (70.9)	50.4 ± 7.5	*t* = 2.017	0.046
	Stage III	43 (29.1)	47.5 ± 8.8		
Postoperative complications	Yes	24 (16.2)	45.3 ± 7.8	*t* = −2.878	0.005
	NO	124 (83.8)	50.3 ± 7.7		
Infectious	Yes	15 (10.1)	42.9 ± 8.8	*t* = −3.536	0.001
	NO	133 (89.9)	50.3 ± 7.6		
Surgical	Yes	9 (6.1)	47.7 ± 7.5	*t* = −0.701	0.484
	NO	139 (93.9)	49.7 ± 8.0		
Clavien I–II	Yes	16 (10.8)	43.6 ± 8.9	*t* = −3.250	0.001
	NO	132 (89.2)	50.3 ± 7.6		
Clavien IIIa or greater	Yes	8(5.4)	47.4 ± 8.0	*t* = −0.776	0.439
	NO	140 (94.6)	49.7 ± 7.9		

BMI, body mass index; ASA, American association of anesthesiologists; HR, hazard ratio; CI, confidence interval; PNI, prognostic nutritional index; CEA, carcinoembryonic antigen; WBC, white blood cell count; AFP, alpha-fetoprotein; CA, carbohydrate antigen. (a: anemia is defined as male HB ≤ 120 g/L and female HB ≤ 110 g/L; b: hypoalbuminemia is defined as ≤ 35 g/L). Abnormal: White blood cell count<4*109/L or >10*109/L.

### 3.2. Clinical characteristics of patients based on preoperative PNI

**Figure 2:**
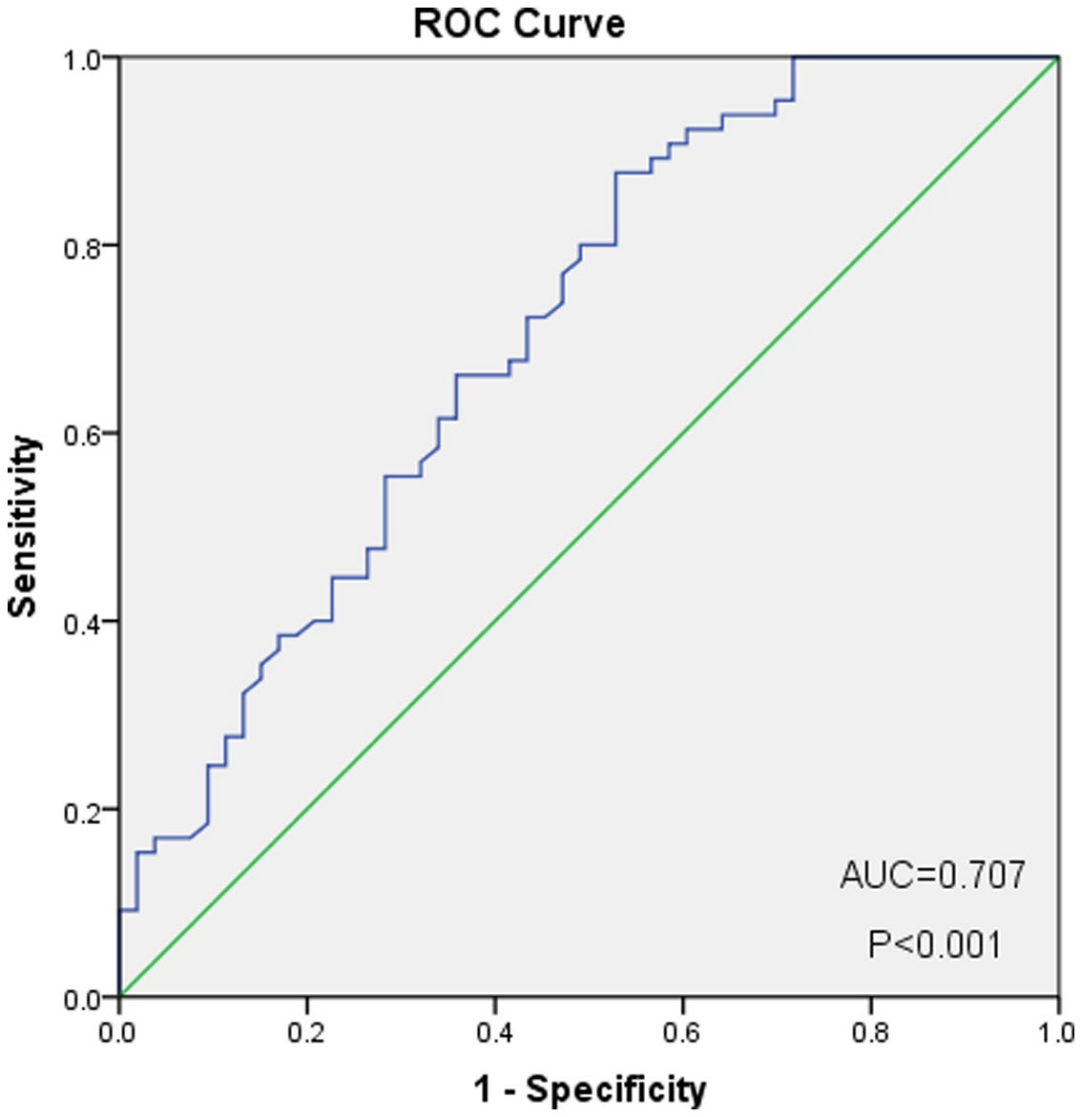
ROC curve for determination of the cut-off value of PNI.

**Figure 3:**
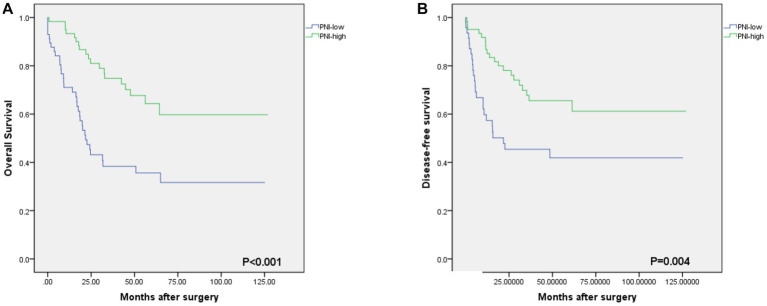
Kaplan–Meier estimates of the overall survival (OS) and disease-free survival (DFS) according to the PNI. **(A)** the OS rate of the PNI-low group was significantly lower than that of the PNI-high group (*p*<0.001). **(B)** the DFS rate of the PNI-low group was significantly lower than that of the PNI-high group (*p* = 0.004).

As shown in Figure 2, the optimal cut-off value of the PNI was 50.0, with sensitivity = 0.646, and specificity = 0.358, corresponding to the maximum Youden index (= 0.349) for the prediction of 5-years OS in the ROC analysis. Based on the cut-off value of the PNI, these patients were divided into a PNI-high group (PNI ≥ 50.0, *n* = 77) and a PNI-low group (PNI <50.0, *n* = 71). Unlike the PNI-low group, the PNI-high group had a significantly higher OS at 1, 3, and 5 years (83.1% vs. 94.8, 60.6% vs. 81.8 and 59.2% vs. 76.6%, respectively, *p* < 0.001) (Figure 3).

The average BMI of the high PNI group was (23.3 ± 2.7) and the low PNI group was (22.1 ± 3.2), with the difference between the two groups being statistically significant (*p* = 0.010). The average hemoglobin level of the high PNI group was (143.1 ± 19.0) and the low PNI group was (119.7 ± 26.1), with the difference between these two groups being statistically significant (*p* < 0.001). The incidence of postoperative complications in patients with high PNI was lower than that of patients with low PNI (*p* = 0.004), while the incidence of infections in patients with high PNI was lower than that of patients with low PNI, and showed a statistically significant difference (*p* = 0.002). The incidence of Clavien I-II events in the low PNI group was higher than that of the high PNI group (*p* = 0.007), while there was no significant difference according to adverse events. Meanwhile, the high PNI groups had tumors of a smaller size (*p* < 0.001), less blood loss (*p* = 0.032), and lower surgical costs (*p* = 0.031) (Table 2).

**Table 2:** Demographic and clinical characteristics of the enrolled patients according to preoperative prognostic nutritional index.

Clinicopathological	PNI-low (*n* = 71)	PNI-high (*n* = 77)	Statistical value	*p* value
Sex			*χ*^2^ = 0.193	0.661
Male	59	66		
Female	12	11		
Age (year)	60.2 ± 8.0	59.8 ± 8.4	*t* = 0.303	0.762
BMI (Kg/m^2^)	22.1 ± 3.2	23.3 ± 2.7	*t* = −2.596	0.010
Tumor location			*χ*^2^ = 0.918	0.632
Upper	28	35		
Lower-middle	27	29		
Mixed	16	13		
CEA (ng/ml)	7.0 ± 14.5	7.4 ± 27.2	*t* = −0.104	0.917
AFP (ng/ml)	56.8 ± 36.2	59.7 ± 42.9	*t* = −0.443	0.658
CA199 (U/ml)	27.5 ± 4.9	28.6 ± 17.4	*t* = −0.710	0.479
CA125 (U/ml)	14.8 ± 14.3	11.2 ± 5.2	*t* = 1.950	0.055
WBC count (*109/L)	6.6 ± 4.7	6.3 ± 3.7	*t* = 0.315	0.754
Hemoglobin (g/L)	119.7 ± 26.1	143.1 ± 19.0	*t* = −6.178	<0.001
Tumor depth			*χ*^2^ = 1.252	0.263
T1, T2	13	20		
T3, T4	58	57		
Lymph node metastasis			χ^2^ = 0.313	0.576
Negative	29	28		
Positive	42	49		
Pathological stage			*χ*^2^ = 0.176	0.675
Stage I, II	40	46		
Stage III	31	31		
Postoperative complications			*χ*^2^ = 8.383	0.004
Yes	18	6		
No	53	71		
Surgical			*χ*^2^ = 0.221	0.738*
Yes	5	4		
No	66	73		
Infectious			*χ*^2^ = 10.950	0.002*
Yes	13	2		
No	58	75		
Clavien-Dindo classification				
Clavien I-II			*χ*^2^ = 8.439	0.007*
Yes	13	3		
No	58	72		
Clavien IIIa or greater			*χ*^2^ = 0.719	0.481*
Yes	5	3		
No	66	74		
Tumor size (cm)	6.4 ± 2.2	4.7 ± 2.0	*t* = 4.757	<0.001
Blood loss (ml)	173.9 ± 129.8	151.3 ± 86.9	*t* = 1.237	0.211
Hospital stay (days)	8.6 ± 3.0	7.7 ± 2.4	*t* = 2.142	0.032
Surgery costs (CNY)	68937.4 ± 18912.1	61994.7 ± 19771.6	*t* = 2.179	0.031

*Using Fisher’s exact test. BMI, body mass index; ASA, American association of anesthesiologists; HR, hazard ratio; CI, confidence interval; PNI, prognostic nutritional index; CEA, carcinoembryonic antigen; WBC, white blood cell count; AFP, alpha-fetoprotein; CA, carbohydrate antigen.

### 3.3. Prognosis of patients based on preoperative PNI

**Figure 4:**
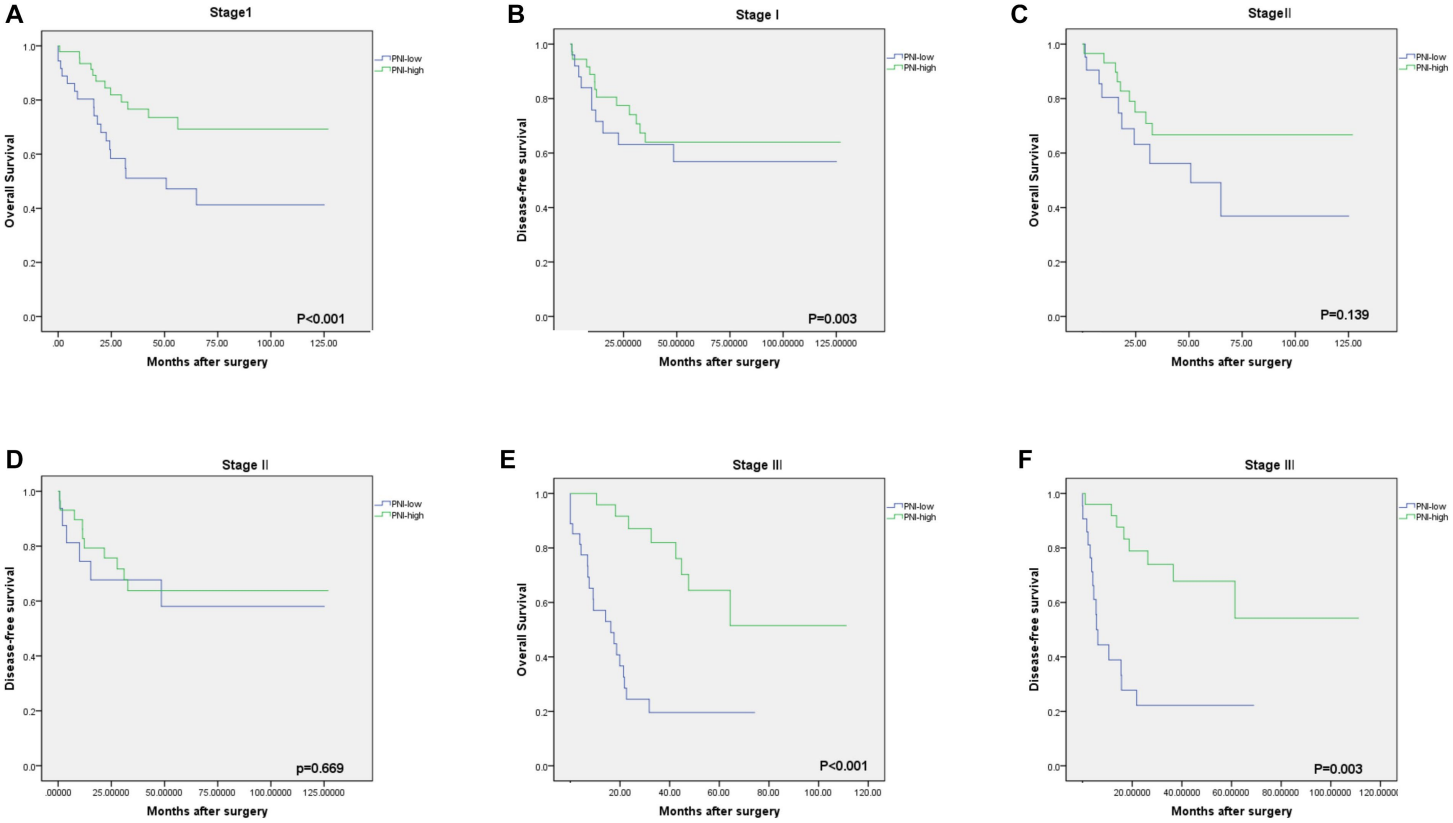
Kaplan–Meier estimates of the overall survival (OS) and disease-free survival (DFS) according to the PNI among patients with stage I [**(A)** OS, *p* < 0.001; **(B)** DFS, *p* = 0.003], stage II [**(C)** OS, *p* = 0.139; **(D)** DFS, *p* = 0.669], stage III [**(E)** OS, *p* < 0.001; **(F)** DFS, *p* = 0.003].

The median OS rate was 30 months (range 6–127 months), and the OS rates at 1, 3, and 5 years were 89.2%, 71.6%, and 68.2%, respectively. The PNI-high group had a significantly better 5-years OS rate (70.5% vs. 42.1%, χ^2^ = 14.745, *p* < 0. 001), compared to the PNI-low group. The 5-year DFS rate was 69.2% in the PNI-high group and 49.0% in the PNI-low group (χ^2^ = 8.374, *p* = 0.004, Figure 3B). Our results showed that a low PNI was associated with a poor OS and DFS in G-NEC patients (Figure 3). Univariate analysis showed that patient age, BMI, anemia, PNI, and pathological stage were associated with prognosis (Table 3). However, only the pathological stage and PNI were independent prognostic predictors (Table 3). Then, we analyzed the predictive value of PNI in patients at different pathological stages. Low PNI was associated with the poor prognosis of patients with stage I and III G-NEC (Figure 4). However, PNI was not associated with a poor prognosis in stage II G-NEC patients (Figure 4).

**Table 3:** Univariate and multivariate Cox regression analysis for overall survival in patients with gastric neuroendocrine carcinoma.

Characteristics	Univariate analysis			Multivariate analysis		
	HR	95% CI	*p*	HR	95% CI	*p*
Gender			0.136			0.257
Male	1.00	Reference		1.00	Reference	
Female	2.170	0.783–6.019		1.952	0.615–6.197	
Age (years)			0.003			0.090
<60	1	Reference		1.00	Reference	
≥60	2.483	1.364–4.518		0.542	0.268–1.099	
ASA score			0.557			0.905
	1	Reference		1.00	Reference	
II–III	1.212	0.637–2.308		0.954	0.438–2.076	
BMI (Kg/m^2^)			0.021			0.074
<18	1	Reference		1	Reference	
≥18	0.389	0.174–0.867		2.649	1.014–6.919	
Tumor size (cm)			0.384			0.151
≤5	1	Reference		1.00	Reference	
>5	1.292	0.725–2.302		1.704	0.824–3.525	
Tumor location			0.387			0.052
Upper	1.00	Reference		1.00	Reference	0.294
Lower-middle	1.425	0.692–2.935		1.446	0.591–3.537	
Multiple	0.960	0.431–2.141		0.816	0.330–2.015	
CEA (ng/ml)			0.128			0.173
<5	1.00	Reference		1.00	Reference	
≥5	1.577	0.877–2.836		0.624	0.317–1.230	
AFP (ng/ml)			0.735			0.456
<7	1.00	Reference		1.00	Reference	
≥7	0.864	0.369–2.021		0.671	0.235–1.915	
WBC count (*10^9^/L)			0.902			0.714
Normal	1.00	Reference		1.00	Reference	
Abnormal	1.037	0.582–1.847		1.140	0.566–2.298	
Hypohemia			0.001			0.461
No	1.00	Reference		1.00	Reference	
Yes	1.259	0.682–2.324		3.730	1.681–8.277	
Tumor depth			0.288			0.374
T1, T2	1.00	Reference		1.00	Reference	
T3, T4	0.288	0.708–3.194		1.551	0.589–4.089	
Lymph node metastasis			0.273			0.507
Negative	1.00	Reference		1.00	Reference	
Positive	0.273	1.389		1.313	0.587–2.936	
Pathological stage			0.001			0.006
Stage I, II	1.00	Reference		1.00	Reference	
Stage III	0.314	0.138–0.714		2.601	1.503–4.504	
PNI			<0.001			<0.001
High	1.00	Reference		1	Reference	
Low	2.876	1.636–5.057		5.955	2.916–12.162	
Chemotherapy			0.837			0.892
Not performed	1.00	Reference		1.00	Reference	
Performed	1.064	0.591–1.915		1.049	0.524–2.102	

BMI, body mass index; ASA, American association of anesthesiologists; HR, hazard ratio; CI, confidence interval; PNI, prognostic nutritional index; CEA, carcinoembryonic antigen; WBC, white blood cell count; AFP, alpha-fetoprotein; CA, carbohydrate antigen.

## 4. Discussion

Studies have shown that, as an important function of the inflammatory response, immunity is closely associated with nutrition and the occurrence and metastasis of tumors (12, 13), and gastrointestinal tumors, in particular, can significantly affect the nutritional (14) and immune status (15) of patients. Therefore, an increasing number of studies have focused on the role of inflammation and nutrition in patients with gastrointestinal tumors. The PNI is calculated based on lymphocyte count and serum albumin level. Lymphocytes release TNF, interferon-γ, and other cytokines, which can inhibit the growth and metastasis of cancer cells. The reduction in its number leads to weakening of the immune function of the body, making cancer cells more prone to immune escape (16, 17), and leading to the poor prognosis of cancer patients (18). Serum albumin is an important nutritional index of the human body. However, in advanced gastrointestinal tumors, there is often insufficient intake and excessive loss, leading to a decrease in albumin levels and an increase in perioperative risk, further affects clinical outcomes (9). Therefore, many studies have found that PNI plays an important role in the development and prognosis of gastrointestinal tumors (19–22). However, no relevant studies have been conducted on G-NEC. At present, there is no unified definition scheme for PNI, but the differences in the data calculated using each different scheme are small, while PNI is obtained through ROC curve analysis (8, 10, 20, 22). The ROC curve analysis showed that 50.0 was the best cut-off point, and patients were accordingly divided into a PNI-high group (PNI ≥ 50) and a PNI-low group (PNI < 50). We found for the first time that PNI can predict the prognosis of patients with gastric neuroendocrine carcinoma and is an independent risk factor for OS of patients with gastric neuroendocrine carcinoma.

The results of our study showed that the mean PNI of patients with a higher pathological stage, as well as anemia and abnormal CEA, was lower than that of patients without such comorbidities. The results may be associated with an increase in tumor biochemical indicators and chronic blood loss when gastric tumors are at the advanced stage. This suggests the necessity of early prevention and supportive therapy (23).

The postoperative complications recorded in our study confirmed that a low immune status before G-NEC surgery was closely associated with postoperative complications. The PNI of patients with postoperative complications was significantly lower than that of patients without complications. At the same time, it was found that the incidence of infections was higher in the low PNI group, which may be associated with a low level of immunity and malnutrition. In the group with complications and Clavien grade I-II, the difference in PNI between the two groups was obvious, but there was no difference in complications in patients with Clavien grade IIIa or greater. This may be because infection-related complications were mostly mild infections, while more serious complications were mostly associated with surgery itself, which is similar to the results of our study.

Currently, several studies conducted on GC have suggested that there is a correlation between PNI and OS, but the mechanism by which it changes according to the stage of the disease is still unknown. Previous reports have found that a low PNI is a predictor of a poor prognosis in patients with stage I and III GC patients, but not at stage II and IV (9). Unlike previous studies, the study by Sakurai (22) found that low PNI could not predict the prognosis of patients at stage III and was a poor predictor of the prognosis of patients at stage I and II. Similarly, we also explored the prognostic value of PNI in patients with G-NEC at different stages. The results showed that in patients with G-NEC stages I and III, a low PNI was significantly associated with poor OS, but a similar result was not obtained for stage II. Since there may be many differences in disease progression between GC and G-NEC, in addition to PNI, the survival rate of patients with stage II G-NEC may also be affected by other clinicopathological factors. Some studies have shown that PNI is of the greatest prognostic value in patients with advanced GC (stage II and III) (21, 24), but it is unclear whether it has the same value in patients with G-NEC. Therefore, more large-scale studies are needed to confirm these results.

Currently, the mechanism of association between PNI and the survival of G-NEC patients is unclear. We speculate that cancer progression is affected by both cancer cells and the immune system. First, albumin can protect cells against tumorigenesis by helping stabilize cell growth and DNA replication (25) and is an independent prognostic factor in ESCC patients (26). Second, lymphocytes inhibit tumor cell proliferation and invasion through cytokine-mediated cytotoxicity (27), in which neutrophils can promote tumor angiogenesis and metastasis, and promote tumor progression by inhibiting *T* cells (28), with an increase in neutrophils associated with poor cancer prognosis (29). Finally, malnutrition and the immune system can interact with each other to promote tumor proliferation and reduce the treatment response (30) and may jointly affect the prognosis of patients with G-NEC.

Our study also has certain limitations. First, since the incidence rate of G-NEC is relatively low, the sample size included in this study is small and the study was retrospective. Therefore, multicenter and large sample clinical studies are needed in the future to obtain more accurate PNI values and better predict the prognosis of patients with G-NEC. Second, due to different sample sizes and patient selection criteria, the optimal PNI values varied between studies, resulting in research bias. Third, many of the G-NEC patients included in our study have just been diagnosed and an evaluation of relevant endocrine indicators was not included. There may be deviations caused by different treatment strategies. In summary, PNI is a simple, practical and effective biomarker, since it can be determined by performing simple blood and liver function tests in patients with G-NEC. For patients with lower PNI values, early intervention may help improve the prognosis and prolong the survival of patients with G-NEC.

## Data availability statement

The raw data supporting the conclusions of this article will be made available by the authors, without undue reservation.

## Ethics statement

This study was approved by the Ethics Committee of First Affiliated Hospital of Air Force Military Medical University. Written informed consent was obtained from the individual(s) for the publication of any potentially identifiable images or data included in this article.

## Author contributions

JW, JL, and HJ: data curation, formal analysis, project administration, software, and writing – original draft. XL: funding acquisition and supervision. XY: investigation. JL and HJ: visualization. JW and XL: writing – review and editing. All authors contributed to the article and approved the submitted version.

## Funding

This work is supported by grants from the National Natural Science Foundation of China (Key Program 82100680) by Gang JI and the Shaanxi Innovation Team (2021-TD-43) by XL and Gang JI.

## Acknowledgments

The authors thank the support of all the medical staff of the Department of Gastrointestinal Surgery, Xijing Hospital.

## Conflict of interest

The authors declare that the research was conducted in the absence of any commercial or financial relationships that could be construed as a potential conflict of interest.

## Publisher’s note

All claims expressed in this article are solely those of the authors and do not necessarily represent those of their affiliated organizations, or those of the publisher, the editors and the reviewers. Any product that may be evaluated in this article, or claim that may be made by its manufacturer, is not guaranteed or endorsed by the publisher.

## Abbreviations

PNI: prognostic nutrition index
G-NEC: gastric neuroendocrine cancer
BMI: body mass index
ASA: American Association of Anesthesiologists
HR: hazard ratio
CI: confidence interval
CEA: carcinoembryonic antigen
WBC: white blood cell count
AFP: alpha-fetoprotein
CA: carbohydrate antigen
OS: overall survival
DFS: disease-free survival
